# A Systems-Pharmacology Analysis of Herbal Medicines Used in Health Improvement Treatment: Predicting Potential New Drugs and Targets

**DOI:** 10.1155/2013/938764

**Published:** 2013-11-28

**Authors:** Jianling Liu, Mengjie Pei, Chunli Zheng, Yan Li, Yonghua Wang, Aiping Lu, Ling Yang

**Affiliations:** ^1^College of Life Science, Northwest University, Xi'an, Shaanxi 710000, China; ^2^Department of Materials Science and Chemical Engineering, Dalian University of Technology, Dalian, Liaoning 116000, China; ^3^Center of Bioinformatics, College of Life Science, Northwest A & F University, Yang ling, Shaanxi 712100, China; ^4^School of Chinese Medicine, Hong Kong Baptist University, Kowloon Tong 999077, Hong Kong; ^5^Lab of Pharmaceutical Resource Discovery, Dalian Institute of Chemical Physics, Chinese Academy of Sciences, Dalian, Liaoning 116000, China

## Abstract

For thousands of years, tonic herbs have been successfully used all around the world to improve health, energy, and vitality. However, their underlying mechanisms of action in molecular/systems levels are still a mystery. In this work, two sets of tonic herbs, so called Qi-enriching herbs (QEH) and Blood-tonifying herbs (BTH) in TCM, were selected to elucidate why they can restore proper balance and harmony inside body, organ and energy system. Firstly, a pattern recognition model based on artificial neural network and discriminant analysis for assessing the molecular difference between QEH and BTH was developed. It is indicated that QEH compounds have high lipophilicity while BTH compounds possess high chemical reactivity. Secondly, a systematic investigation integrating ADME (absorption, distribution, metabolism, and excretion) prediction, target fishing and network analysis was performed and validated on these herbs to obtain the compound-target associations for reconstructing the biologically-meaningful networks. The results suggest QEH enhance physical strength, immune system and normal well-being, acting as adjuvant therapy for chronic disorders while BTH stimulate hematopoiesis function in body. As an emerging approach, the systems pharmacology model might facilitate to understand the mechanisms of action of the tonic herbs, which brings about new development for complementary and alternative medicine.

## 1. Introduction

Many diseases, particularly the chronic diseases such as rheumatoid arthritis, tuberculosis, or multiple myeloma, may lead to decreased immune system function, deficiency of vital energy and blood. Patients usually displayed pale complexion, spontaneous sweating, loss of appetite, shortness of breath and even hypothermy, cold limbs syndrome, dizziness, susceptible to more severe diseases. Throughout recent years, there are growing interests in Traditional Chinese Medicine (TCM) from natural sources as a feasible alternative therapeutic agent for the prevention of these chronic disorders or to improve health condition, boost immunity and keep the body in balance to lowers disease risk.

In many Asian countries, two kinds of tonic Chinese herbs, so called Qi-enriching herbs (QEH) and Blood-tonifying herbs (BTH), are widely used in daily life to improve health for thousands of years. QEH enhance physical strength and endurance, strengthen the immune system and restore normal well-being. BTH are used to nourish the blood, invigorate circulation, regulate the menstrual cycle and are used for various patterns of blood deficiency. For QEH, ***Ginseng*** has been used medicinally in the Far East for several millennia and has been believed to enhance well-being as a representative qi tonic drug, it is beneficial for immune function, central nervous system function, cardio- and cerebro-vascular functions [1]. Other indicative herbs for the treatment of qi deficiency are ***Codonopsis pilosula***, ***Panax quinquefolium***, ***Radix pseudostellariae***, ***Radix astragali***, ***Rhizoma atractylodis macrocephalae***, ***Dioscorea opposite***, and ***Radix glycyrrhiz,*** which have always being prescribed in many herbal formulae. For example, Huang Qi Liu Yi Tang (HQLYT, composed of ***Radix*** ***glycyrrhiz*** and*** Radix astragali***) is widely used in daily foods [2]. For BTH, ***Angelica sinensis*** is considered as a blood tonic. It is useful for replenishing and invigorating blood, and relieving pain, resulting in treatment of menstrual disorders, menorrhagia and hematopoiesis disorders [3]. Except for ***Angelica sinensis***, other herbs which have effect of blood-tonifying are ***Radix paeoniae alba***, ***Polygonum multiflorum***, and ***Rehmannia glutinosa***. Clinically, Si-Wu-Tang, including three blood-tonifying medical plants, that is, ***Radix paeoniae alba***, ***Angelica sinensis*** and ***Rehmannia glutinosa***, is one of the most widely used TCM formulae in China and Japan (Japanese name, Shimotsuto). It has been reported to possess anticoagulant activities and exhibit hematopoiesis, vasodilation and alleviate dysmenorrhea [4].

Traditionally, qi-enriching herbs were used to treat weakness, loss of appetite, fever, fatigue and uterine prolapse [5]. Nowadays, the qi-enriching herbs are widely used in modern medicine as an immune enhancer [6–9], and to prevent the common cold, influenza, and chronic hepatitis. Emerging evidence further suggests that qi-enriching herbs are useful as an alternative treatment for patients whose immune systems have been compromised either by cancer [10] or AIDS [11]. Recently, it is also proposed that the qi-enriching herbs may act as a possible antioxidant in patients [11, 12] with severe heart and liver diseases, leading to symptom relief and improvement in organ function [13]. For blood-tonifying herbs, they have been reported to exhibit pharmacological actions such as replenishing and invigorating blood, augment blood of coronary artery, anti-acute myocardial ischemia [14], anti-platelet agglutination, anti-thrombogenesis [15], activation of fibrinolysis, decreased blood fat [16], improvement of the blood hypervisicosity syndrome, depressed blood pressure, anti-artherosclerosis, and heart or liver protection [17, 18]. However, owing to the complexity of the basic theory of TCM, the molecular basis of QEH and BTH and the mechanisms of action are still a mystery. Hence, the qi-enriching and blood-tonifying natures in health improvement treatment are far from sufficient, approximately a “black” box. Fortunately, the fast development in “omics” technology and systems biology has provided a chance to deep understand the mechanisms concerning with TCM. The systems-based analysis approach can allow for TCM drug action to be considered in the context of the whole system, thus acceleration the uncovering the molecular mechanisms related to the therapeutic efficacy from a systematic point of view [19]. In our previous work, we have successfully used the systems-based method to investigate the mechanisms of action of TCM with application to cardiovascular diseases [20–22].

In the present work, for the first time we shed light on the mechanism of action of the QEH and BTH in health improvement treatment. The proposed model integrates the oral bioavailability screening, pattern recognition, discrimination analysis, drug targeting and network pharmacology analysis. The mechanisms of action of tonic herbs with a holistic way to improve health condition as well as treating disease were deeply uncovered. Taken together, this study extends our understanding of the molecular/systems mechanisms underlying tonic herbs activity observed in oriental medicine, and also be helpful to bring about a new opportunity to the development in the complementary and alternative medicine.

## 2. Materials and Methods

**Figure 1:**
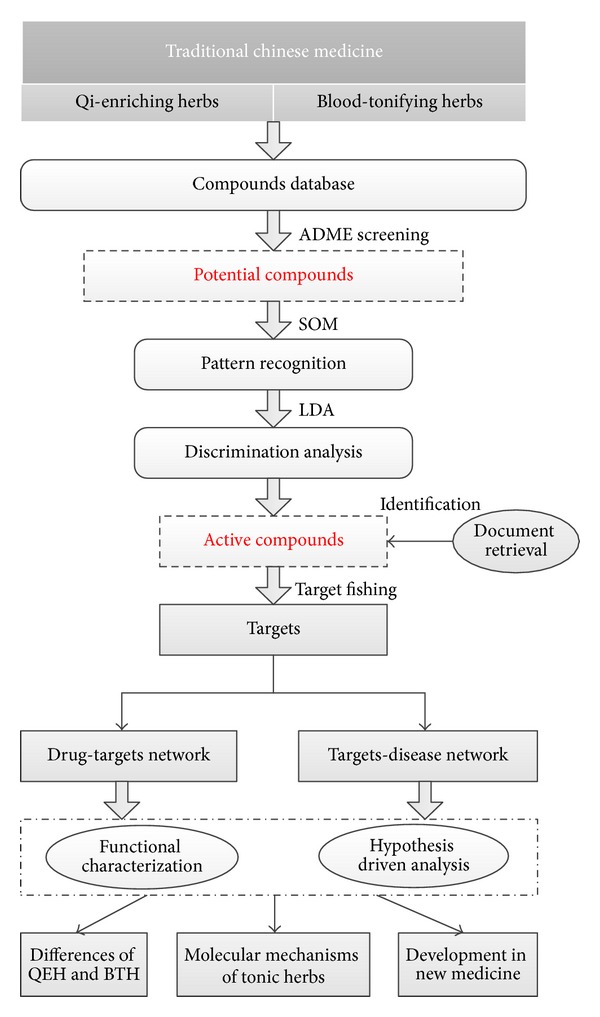
Flowchart of the model building.

Our protocol includes four main steps: (1) obtainment of QEH and BTH and building the ingredient database; (2) screening the potential active compounds with Oral Bioavailability and Druglikeness analysis; (3) generation of the chemical and pharmacological features of the herbs; (4) building of drug-target networks; and (5) systems pharmacology analysis.  Figure 1 depicts the flowchart of the whole experimental procedure.

### 2.1. Chemical Database and Descriptor Calculation

Traditional Chinese Medicine has a history of 1000 years fighting against Qi Deficiency Syndromes (QDS) and Blood Deficiency Syndromes (BDS) and has accumulated numerous of prescriptions and clinical practices. In this work, a large-scale text mining approach [23] was applied to obtain the indicative herbs which were used for the treatment of QDS and BDS. Finally, a total of 12 herbs including 8 in QEH (*Ginseng, Codonopsis pilosula, Panax quinquefolium, Radix pseudostellariae, Radix astragali, Rhizoma atractylodis macrocephalae, Dioscorea opposita, Radix glycyrrhiz *resp.), 4 in BTH were collected (*Radix paeoniae alba, Angelica sinensis, Polygonum multiflorum, Rehmannia glutinosa*).

All chemicals of each herb were retrieved from our in-house developed database: The Traditional Chinese Medicine Database and Systems Pharmacology Analysis Platform (http://tcmspnw.com/). This database involves about 500 herbals which have been registered in the Pharmacopoeia of People's Republic of China [24]. To the largest extent, 31,871 organic molecules have been collected in this database. In the present work, 1498 compounds were compiled into our data set, including 170 in *Ginseng*, 128 in *Codonopsis pilosula*, 140 in *Panax quinquefolium*, 98 in *Radix pseudostellariae*, 118 in *Padix astragali*, 66 *Phizoma atractylodis macrocephalae*, 69 in *Dioscorea opposita*, 279 in *Radix glycyrrhiz, *86 in *Rehmannia glutinosa*, 111 in *Radix paeoniae alba*, 65 in *Polygonum multiflorum*, 168 in *Angelica sinensis* respectively. All the compounds were kept as mol2 format and their information was provided in Tables S1 and S2 (Supporting Information). See supplementary martial available online at http://dx.doi.org/10.1155/2013/938764.

In the present work, the data set was preprocessed to remove the duplicate compounds in the BTH and QEH to reduce the redundancy of the data. The molecular descriptors were calculated using DRAGON 5.4 program (http://www.talete.mi.it/index.htm). As a result, 1664 descriptors were calculated for each compound from 20 molecular descriptor blocks: constitutional descriptors, topological descriptors, two-dimensional (2D) autocorrelations, molecular properties et al. Based on the multicollinearity correlation analysis [25] and mutual information method [26], the redundant descriptors were removed and 135 descriptors finally remained for further analysis.

### 2.2. Active Compounds Screening

Screening of the active compounds comprises Oral Bioavailability (OB) screening and Drug-likeness (DL) prediction. OB, represents the rate and extent to which the active ingredient or active moiety is absorbed from a drug product and becomes available at the site of action [27]. Here the OB screening was performed by an in-house system OBioavail1.1 [28] and the compounds with OB ≥ 30% were kept in the database. The threshold determination was based upon careful consideration of the followings: (1) Extract information from QEH and BTH as much as possible using the least number of chemicals. (2) keeping consistency with the reported pharmacological data. In addition, for the purpose of filtering out the drug-like molecules, we have developed a database-dependent model to discriminate between drug-like and nondrug-like chemicals using the Tanimoto coefficient [29]. In this work, the compounds with DL ≥ 0.2 were selected as the Drug-like Compounds and the threshold is the average DL for DrugBank database. Numerous studies have shown that although many compounds have low OB, but have strong pharmacodynamic effects at a very low dose [30, 31]. In order to get more comprehensive compounds for in-depth study, the compounds which exceed either OB or drug-likeness screening were regarded as the candidate compounds for BTH and QEH.

### 2.3. Pattern Recognition

In order to visualize the qi- and blood-relevant compounds in a 2D space representing the structural information encoded in the molecular descriptors, the self-organizing map (SOM) was used. SOM is a type of artificial neural network trained by unsupervised machine learning approach to produce a two-dimensional, discretized representation of the input space of the training samples [32]. By assigning each input vector to the neuron with the nearest feature vector, the SOM clustering algorithms attempt to organize unlabeled in-put vectors into clusters,therefore points within a cluster are more similar to each other than vectors belonging to different clusters [33]. that is, similar clusters are (usually) grouped together. And  *U*-matrix was used for visualization of data. The  *U*-matrix value of a particular node represent the average distance between the node and its closest neighbors [34].

The self-organizing map simulations were carried out using internally developed C-language program. The SOM network of dimension  10 × 10  was applied, which enabled one to map objects into 100 positions. Similar objects were mapped into the same position (*x*, *y*  coordinate in a SOM).

### 2.4. Discrimination Analysis

In order to provide a formula which is simple to apply, easy to interpret and always gives good results for solving qi-blood compounds classification problem, a stepwise linear discriminant analysis (S-LDA) was applied to build model. In this work, the training sets were used to explore the most important variables related to the biological activities, while the test set was used to validate the performance of the model. The whole data set was randomly split into training and test sets in a ratio of about 75% : 25%. The structural applicability domain for the model was obtained by visualizing the SOM pattern to select the typical areas which represent the qi or blood compounds.

### 2.5. Target Identification and Validation

In order to obtain the targets for drugs, we have developed a robust *in silico* model [35] to identify potential targets for a given chemical molecule, and the criteria with SVM score ≥ 0.8 and RF score ≥ 0.7 was applied in this work. In addition, the targets were also predicted by the SEA method (http://sea.bkslab.org/), which is only limited to the human targets. Moreover, the targets also retrieved from TTD (http://bidd.nus.edu.sg/group/ttd/), STITCH (http://stitch.embl.de/) and HIT (http://lifecenter.sgst.cn/hit/welcome.html), all of them are resources to explore known and predicted interactions of chemicals and proteins. And all the obtained targets were retrieved from TTD (http://bidd.nus.edu.sg/group/ttd/) and Uniprot (http://www.uniprot.org/) to explore the interactions of chemicals and proteins.

### 2.6. Compound-Target-Disease Networks Construction

In order to explore the potential therapeutic effect of certain drugs and suggesting novel drug targets. The Candidate Compounds, Potential Targets and Diseases information were used to build the Compound-Target-Disease networks (C-T-D network). In the graphical network, each component such as compound, protein and disease is described by node, and the interactions are encoded by edges. The C-T-D network was produced through two steps: (1) the drug-target connections were generated by linking the candidate compounds with all their potential targets. (2) The potential targets were extracted from the TTD (http://bidd.nus.edu.sg/group/ttd/) and Uniprot (http://www.uniprot.org/) for their diseases information. All the networks were generated by Cytoscape 2.8.1 [36].

## 3. Results and Discussion

The complexity of the chemical components makes it extremely difficult to understand the mechanisms of action for herbal medicines from a molecular or systems level. How to understand the TCM system as a whole (i.e., the external signs) and the internal changes in the relevance has, thus, become the “bottleneck” of modern TCM study. Therefore, in addition to the specific focus on the issue of Qi-Blood, the present work also attempts to interpret the abstraction theory of TCM through relatively simple two kinds of botanical drugs. Here, we have constructed an integrated system combining ADME screening, pattern analysis, drug targeting, and network pharmacology to investigate the systems-level mechanisms of action for the QEH and BTH.

### 3.1. Active Ingredients Identification

In most cases, herbs are orally administered. It is believed that most compounds in the mixture fail to reach to the cellular targets as they lack appropriate pharmaceutical properties, such as favorable oral bioavailability [37]. Therefore, the OB screening together with the drug-likeness evaluation should be indispensable to determine whether a compound is pharmaceutically active in a complex herb mixture. We found that 849 compounds which account for 69% of all the 1068 chemicals in the BTH have satisfactory oral bioavailability (OB ≥ 30%) or favorable drug-likeness property (DL ≥ 0.2), and 361 accounting for 67% of all the 430 chemicals in the QEH have OB ≥ 30% or DL ≥ 0.2. The details are provided as following.

#### 3.1.1. Qi-Enriching Herbs

***Rhizoma atractylodis macrocephalae***. 47 compounds were obtained with good OB or favorable DL from this herb. For example, atractylenolide II and atractylenolide III are the main pharmacologically active constituents with the ideal OB values (atractylenolideII: OB = 47.50%; atractylenolideIII: OB = 68.11%). In ***Radix astragali***, 101 chemicals have good OB or DL features, among which tangshenoside I exhibits significant antibacterial activity and lowering blood sugar function. As for ***Codonopsis pilosula***, 86 of 128 compounds with good OB or DL were collected, tangshenoside I, as one of the major component, has the OB of 68.77%. 108 compounds were filtered out from the total 170 chemicals from ***Ginseng***. For example, ginsenoside Rg1 has been demonstrated possessing significant immunomodulatory activity and ginsenoside-Rb2 which can inhibit lung tumor metastasis. 61 compounds from ***Dioscorea opposite*** include ergosterol, one of major ingredients in this herb, were obtained. In ***Radix glycyrrhiz***, 255 compounds possess desirable OB or DL properties. Interestingly, all the major components including isoliquiritigenin, licochalcone A, ononin were all screened out, which have been proved to have anti-microbial activity and anti-inflammatory activity. As for ***Radix pseudostellariae***, 72 compounds are potential active components, and 86 compounds accounting for 87.7% of all the compounds in ***Panax quinquefolium*** also show ideal pharmacokinetic properties such as Papaverine (OB = 64.04% and DL = 0.38).

#### 3.1.2. Blood-Tonifying Herbs

87 compounds were collected from ***Radix paeoniae alba***, such as compound albiflorin, one of the major ingredients, has the ideal OB and DL properties (OB = 49.12% and DL = 0.79). As for ***Polygonum multiflorum***, 59 compounds are potentially active. In ***Angelica sinensis***, in the obtained 109 active molecules, ferulic acid (OB = 54.01%) is the main ingredient, which can promote hematopoietic progenitor cell proliferation and differentiation [38]. 69 compounds in ***Rehmannia glutinosa*** are potentially active, such as Rehmannioside A (OB = 25.94% and DL = 0.87).

### 3.2. Clustering Analysis

**Figure 2:**
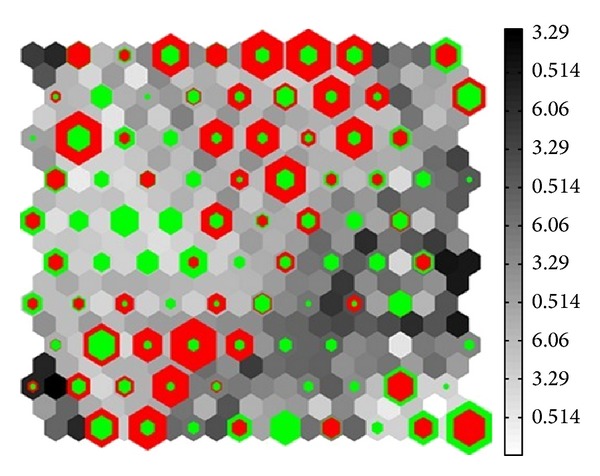
Visualization of the  10 × 10  SOM of qi versus blood compounds using  *U*-matrix. Every square corresponds to a map neuron in the same position. The green and red colors represent qi, blood compounds, respectively.

1210 candidate compounds (849 qi-enriching compounds and 361 blood-tonifying compounds) which meet either OB or DL screening criteria were used for further clustering analysis. The SOM method was applied here to place the prototype vectors (135 descriptors) on a regular low-dimensional grid by using the  *U*-matrix technique. A  10 × 10  map was obtained as shown in Figure 2. The green and red nodes represent qi-enriching compounds and blood-tonifying compounds, respectively. Surprisingly, these two kinds of compounds were well clustered in particular regions in the map. Figure 2 depicts that the qi-enriching compounds (in green color) distributed irregularly throughout the map in several subgroups, whereas the blood-tonifying compounds (in red color) mainly concentrated on the upper right and bottom left part of the map. The areas occupied by the qi-enriching compounds are relatively large and scattered compared to those of the blood-tonifying compounds, reflecting the broad molecular diversity and large chemical space for qi-enriching compounds. In addition, several overlaps were also observed as shown in mixed-up colors (the red-green), indicating that these nodes were occupied by qi-enriching compounds and blood-tonifying compounds. On the assumption that structurally similar drugs tend to bind similar proteins, drug chemical structure information is a good indicator for drug biological activity [39]. The herbal compounds with the similar structure can cluster together, as a consequence, the compounds in each well-separated region may have similar mechanisms of actions. Thus, we deduce that qi-enriching herbs have a huge potent to hit massive targets and treat a variety of ailments ultimately. Conversely, the region of the blood-tonifying compounds is relatively focused, which, to some extent, shows that the blood-tonifying compounds share more chemical and biological similarities with each other. Thus, we deduced that targets and curative effect of blood-tonifying herbs are relatively single/centralized compared to the qi-enriching herbs.

### 3.3. Discrimination Model

Information on molecular properties of qi and blood herbs is required in order to assess the category of chemical compounds from various herbs. However, the experimental determination of such a large quantity of molecules is time-consuming and expensive. There is a preference to establish rapid, sensitive and low-cost computational models rather than the traditional and expensive experimental approaches for predicting the qi or blood chemicals from the knowledge of molecular structures [40].

LDA algorithm formulizes the relationship between variables and response in clear numbers or coefficients. This endows their usage and reliability, particularly for explanation of the mechanism. So, a model generated by LDA algorithm is beneficial and simple to be interpreted with inductive theoretical descriptors, as it allows interpreting individual descriptors into molecular activity, and each descriptor could be seen by the magnitude and sign of its discriminant coefficient.

In this work, from the large training set of compounds, the stepwise LDA was applied as a variable selection strategy in order to select the best combination of those descriptors most relevant to activities. Thus the study outlines the details of the one well performed model to estimate the nature of qi-blood herbs which are reported in the following equation. Descriptors are written in decreasing order of the significance, based on the absolute size of their standardized regression coefficients.

The best LDA model for the qi-enriching herbs and blood-tonifying hers were generated using a training set of 908 compounds and 302 external test compounds with 5 molecular descriptors. The average classification accuracies of the leave-one-out cross-validation are 83.3% (*Q*_cv_) for the training set and 82.2% (*Q*_ex_) for the testing date, indicating that the S-LDA model is highly reasonable

(1) D=0.622×MlogP−0.156×Mor27m+0.982 ×VEA1−0.046×H-046 −0.966×HATSOe−4.644,Ntr=908,   Nte=302,   Qcv=83.3%,Qex=82.2%, SEqi=82.5%,   SPqi=15.7%,SEblood=84.3%, SPblood=17.5%,

where Ntr and Nte are the number of compounds in the internal training and external test sets individually,  *Q*_cv_  and  *Q*_ex_  are the overall classification accuracy of internal LOOCV and external test sets respectively. And the model was subsequently validated by SE (sensitivity) and SP (specificity) [41] which further demonstrate the reasonability of the obtained model.

For the optimal LDA equation, the standardized coefficient reveals that the most significant descriptor is MlogP, which indicates the penetration/lipophilicity of a compound to form non-covalent interactions with its environment, to dissolve and persist in water or in a lipidic environment, or to permeate the phase interfaces. Normally, larger MlogP indicates a stronger ability of the chemical to permeate cell membrane of an organism. In (1), the MlogP takes a positive weight, which indicates that for this function increasing values of MlogP correlate with increasing the possibility of qi-enriching action. Mor27m is, as the second most important descriptor, which is a 3-D MoRSE descriptor (3-D Molecular Representation of Structures based on Electron diffraction). Mor27m descriptor is calculated by summing up the atomic weights viewed by different angular scattering functions and weighted by atomic electronegativity. The reactivity descriptor indicates the compound's ability to interact with its surrounding molecules and to form chemical bonds. It takes a negative weight in the equation, indicating that increased values of Mor27m are correlated with increasing action rating of ablood-tonifying compound. Further analysis of the Mor27m values, we surprisingly found that a compound is very likely to be qi-enriching compounds when its Mor27m is less than −1, as indicated by the fact that 74.6% compounds in all the test qi-enriching 366 molecules have the Mor27m values less than 1. If Mor27m is larger than −1, the compound is likely belonging to blood-tonifying compounds (62.9% in the all the test 288 blood-tonifying compounds larger than −1).

VEA1 indicates the eigenvalue coefficient sum from the adjacency matrix of a molecule. H-046 represents the number of hydrogen atoms attached to a sp3 carbon. The role of this descriptor is to basically show the composition features of the skeleton of a compound. It is interesting to find that there are 258 (70.49%) compounds which have larger than 5-valued SHCsats for qi compounds and 197 (68.4%) compounds which have less than 5-valued SHCsats for blood compounds. All this indicates that compounds with nonsaturated C are potentially qi-enriching compounds. HATS0e means leverage-weighted autocorrelation of lag 0, weighted by atomic Sanderson electronegativity. More electronegative and labile atoms will have larger positive field density, which indicates higher chemical reactivity of a molecule. The presence of this electronegativity in a molecule tends to indicate the role of the chemical reactivity on idenfication of qi-enriching and blood-tonifying compounds. Yet the three groups play totally different roles in affecting the qi-blood compounds, with H-046 and VEA1 contributing positively but HATS0e negatively to the structural model.

Generally, this equation contains two types of descriptors: those describing molecular lipophilicity, shape, bulk properties and those reflecting chemical reactivity. This indicates the importance of taking lipophilicity and chemical reactivity into account for the prediction of qi-enriching and blood-tonifying compounds. In general, qi-enriching compounds have characteristics of high lipophilicity while blood-related compounds possess high chemical reactivity.

### 3.4. Compound-Target-Disease Networks

The active compounds applicability domain for the C-T-D Network was obtained by  (1)  visualizing the SOM pattern to select the typical areas which represent the qi-enriching or blood-tonifying compounds, (2) remaining the correct qi or blood classification of compounds after discrimination analysis and (3) picking out effective compounds in accordance with the literature.

**Figure 3:**
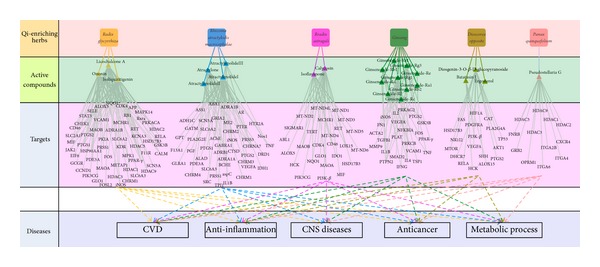
Target network and functional enrichment of the QEH. A target network of each herb was uniquely identified by mapping the possible targets of each herb into QEH-specific molecular network. The functions for the target networks were divided into 5 classifications. These enriched biological functions are associated with qi-enriching. Diagram frame labeled in pink color denote QEH, diagram frame labeled in green color denote the active compounds of the each QEH, diagram frame labeled in purple color represent targets of active compounds, and diagram frame labeled in blue color represent functions for the target networks. The effective molecules of ***Codonopsis pilosula*** and ***Radix pseudostellariae*** are contained in ***atractylodes*** and ***Ginseng***, so target analysis no longer mentions ***Codonopsis pilosula*** and ***Radix pseudostellariae***.

**Figure 4:**
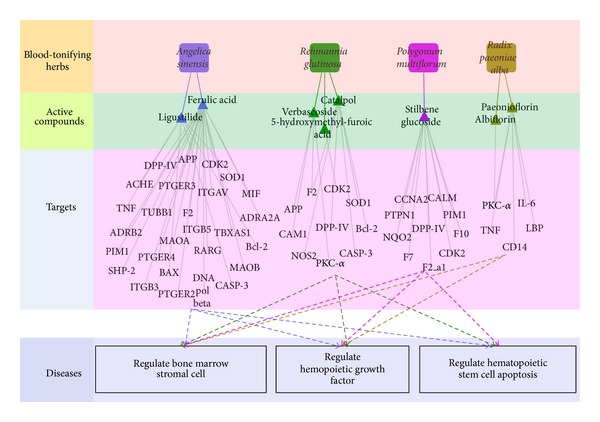
Target network and functional enrichment of the BTH. The functions for the target networks were divided into 3 classifications. Diagram frame labeled in color pink, green, purple and blue denote BTH, active compounds of the each BTH, targets of active compound and functions for the target networks, respectively.

The availability of large phenotypic and molecular networks provides a new opportunity to study the association between compounds and proteomic datasets. Surprisingly, correlating the drug-target map with a target-disease map suggests that many drugs are not acting on a protein most directly implicated in a disease but rather acting at one or two degrees of separation, at proteins which are linked to the disease genes. Thus, we propose that the drug-target network associated with disease may be an effective strategy to explore the potential therapeutic effect of certain drugs and therefore suggests novel drug targets. After deleting the 8 compounds with no targets, the resultant “Candidate Compounds”, “Potential Targets” and all their “Disease” were applied to generate a graph of Compound-Target-Disease interactions, in which a compound and a target are connected to each other if the protein is a Candidate Target of the Compound, the disease is linked to the Candidate target if the target has relationships with the disease, giving rise to a “C-T-D Network” (Figures 3 and 4).

#### 3.4.1. QEH

Constructing the target protein network for a specific disease or drug could help improve understanding the mechanism of certain drugs on specific diseases. In our study, there are 146 potential targets hit by 31 active compounds in all 202 targets, which have been annotated to have significant relationships with the pharmacologic effects of improving health. Detailed information is given in (Table 1).

*Anti-Inflammation.* Inflammation is the body's attempt at self-protection; the aim being to remove harmful stimuli, which ultimately leads to the restoration of normal tissue structure and function. A normal inflammatory response is self-limiting and involves the down-regulation of pro-inflammatory protein expression, increased expression of anti-inflammatory proteins, and a reversal in the vascular changes that facilitated the initial immune cell recruitment process. Qi-enriching herb *Radix astragali* extracts have been known to have strong anti-inflammatory effects by increasing white blood cells, multinuclear leukocytes and IgM in mice [42]. *Ginseng* also has immune-enhancing effects, it increases the function of the reticuloendothelial system and the total amount of IgM [43].

In this work, 15 targets have significant relationship with the inflammatory processes. For example, MIF (Macrophage migration inhibitory factor) participates in inflammation-mediated rheumatoid arthritis (RA) and systemic lupus erythematosus [44], which is hit by 3 active compounds in *Radix astragali* and *liquorice*. Interestingly, for these 3 compounds, It has been demonstrated that isoliquiritigenin (ISL, *liquorice*) induces HO-1 expression by activation of ERK1/2 MAPK and ISL-induced HO-1 expression which is considerably associated with the LPS-induced NO and TNF-a production in murine macrophages. Thus, ISL is an effective HO-1 inducer, capable of inhibiting macrophage-derived inflammation. Calycosin (*Radix astragali*) also has been reported to reduce AGEs-induced macrophage migration and adhesion to endothelial cells via estrogen receptor-ERK1/2-NF-*κ*B pathway to achieve anti-inflammation effect [45]. Molecules atractylenolideI, II, III, ginsenoside, licochalcone A and ononin were found to target iNOS (Nitric oxide synthase, inducible), which is involved in the host defense in bronchial epithelium and acts as an inflammatory mediator in pathological states [46]. Furthermore, PTGS1 (prostaglandin G/H synthase 1) and PTGS2 participate in inflammation-mediated cytotoxicity and neuronal death [47], which is hit by atractylenolideI, ononin, licochalcone A, isoliquiritigenin, Diosgenin-3-O-beta-D-glucopyranoside and ginsenoside. Among these compounds, licochalcone A has been reported to inhibit prostaglandin biosynthesis in lipopolysaccharide (LPS)-induced mouse macrophage cells to achieve anti-inflammation effect [48]. Control of those targets related to inflammation will lead to the improvement of immunoreaction, inhibition of inflammatory process and prevention damage of the inflammatory factor. In addition, collaboration between anti-inflammatory and immunostimulatory effects may contribute to the best immune response, which might be the integrated actions of compound mixtures from various medicinal herbs [6, 10, 49].

*Cardiovascular System Diseases. *Cardiovascular disease (CVD), a leading cause of deaths worldwide, refers to any disease that affects the cardiovascular system, principally cardiac disease, vascular diseases of the brain and kidney, and peripheral arterial disease [50]. The causes of cardiovascular disease are diverse but atherosclerosis and/or hypertension are the most common [51]. In traditional medicine, qi-enriching herbs have also been widely used for treatment of renovascular and cardiovascular diseases, and hence frequently constitute a major ingredient of polyherbal formulations for cardiovascular diseases [24]. Clinical studies have demonstrated that *Ginseng* has a positive inotropic effect on the hearts of dogs, rabbits and other animals. It increases the contractility of the heart, slightly increases blood pressure, and constricts blood vessels. It has shown beneficial effects in treatment of shock and arrhythmia [52]. *Radix glycyrrhiz* also has desirable pharmacological effects on preventing atherosclerosis, increasing resistance of low-density lipoprotein (LDL) to atherogenic modifications, reducing plasma lipid levels, and decreasing systolic blood pressure [53], and even showing certain impacts on antiarrhythmic, and brain ischemia, and so forth [54].

Interestingly, inspection of the network identifies 28 proteins, which have significant relationships with the cardiovascular diseases. For example, HDAC1 (Histone deacetylase 1) plays a crucial, nonredundant role in cardiomyocyte differentiation and maturation and has been used in cardiovascular therapeutic applications [55]. TSP1 (thrombospondin-1) has been uncovered critical roles in acute regulation of cardiovascular dynamics, hemostasis, and immunity by interfering with the NO-mediated vasodilatory pathway [56]. And *Ginseng*-Re which interacts with TSP1 possesses many beneficial effects on cardiovascular system. G-Re has negative effects on cardiac contractility and autorhythmicity, which accounts for its antiarrhythmic effect. In addition, G-Re also exerts antiischemic effect and induces angiogenic regeneration [57]. Molecules ononin, ginsenoside-Rb, atractylenolide I, and diosgenin-3-O-beta-D-glucopyranoside were found to target the VEGF (vascular endothelial growth factor), which is the key regulator in vascular biology [58]. VEGFs have received much attention regarding their potential therapeutic use in cardiovascular medicine, especially for therapeutic vascular growth in myocardial and peripheral ischemia [59]. We found many isoflavonoids are connected to VEGFA. For example, ononin has markedly positive effect in prevention of cardiovascular diseases [60]. All these findings suggest that qi-enriching herbs might regulate the whole cardiovascular system by a complex protein-protein interaction network, and ultimately cure of the cardiovascular disease.

*Metabolic Process. *The most common metabolic disorders include diabetes, obesity, adrenoleukodystrophy, glucose galactose malabsorption and phenylketonuria, and so forth. The goal of treatment is to reduce risk of heart disease and diabetes, some people may benefit from daily low-dose aspirin which can address symptom relief. It has been suggested herb *Ginseng* has anti-hyperglycemia, and anti-obesity effects. The mechanism of action is related to insulin sensitization, insulin secretion, *β*-cell protection, thermogenesis and antioxidantion, thus it is a promising herbal remedy in the treatment of metabolic syndrome [61].

It was found that 13 active compounds connected 33 targets which are concerned with metabolic diseases, so through the modulation to these proteins, these compounds may relieve pathogenic risk, finally slow the progression of the disease condition and reduce the symptoms. For example, M(3) mAChR (Muscarinic acetylcholine receptor M3) plays a key role in regulating many important metabolic functions, M(3) mAChRs are essential for maintaining proper insulin release and glucose homeostasis, it also suggested that strategies aimed at enhancing signaling through beta-cell M(3) mAChRs might be beneficial for the treatment of type 2 diabetes [62]. Proteins PPAR-gamma (Peroxisome proliferator-activated receptor gamma) have been confirmed to be closely related to disordered glucose homeostasis, adipogenesis, lipid metabolism, and blood pressure regulation [63]. CALM (Calmodulin) may be involved in prostaglandin metabolism; it activates phospholipase A2, leading to the synthesis in platelets of PGHz and thromboxane. The influence of CALM on prostaglandin metabolism obviously has relevance to the role of platelets and the vascular endothelium in haemostasis [64, 65]. FAS (Fatty acid synthase), the sole mammalian enzyme capable of de novo fatty acid synthesis, is associated with poor prognosis in breast and prostate cancer, is elaborated into the blood of cancer patients, and its inhibition is selectively cytotoxic to human cancer cells [66]. Thus, the compounds interacting with these receptors may be key players in the relief of metabolic syndrome.

*Anticancer Process*. Currently experiments have been shown that herbs play its anticancer role by inducing apoptosis and differentiation, enhancing the immune system, inhibiting angiogenesis, reversing multidrug resistance (MDR), and so forth. Clinical trials also demonstrated that TCM could improve survival, increase tumor response, improve quality of life, or reduce chemotherapy toxicity [67].

An inspection of our network found that flavonoids (licochalcone A) saponins (ginsenoside, dioscin) and atractylenolide have strong interactions with 27 tumor-associated targets. In vitro/in vivo studies have established an association between flavonoid-induced modulation of protein kinase and MMP activities with apoptosis, cellular proliferation and tumor cell invasive behavior [67]. Ginsenoside binding to TNF (Tumor necrosis factor) has been demonstrated increasing effects of antiproliferative on two human colorectal cancer cell lines [68]. In addition, Cdk4 inhibition has been shown to induce potent G1 arrest in vitro and tumor regression in vivo [69]. So through the modulation to these proteins, the QEH may achieve the antineoplastic curative effect, thus improving the health conditions when combined with Western medicine for treating tumor diseases.

*Central Nervous System Diseases. *Central Nervous System (CNS) diseases affect millions of human beings. Despite aggressive research, patients are still suffering from fatal and/or debilitating CNS diseases. The low toxicity, moderate cost and natural abundance make TCM an attractive substance for treatment of CNS.

As can be seen from our network, proteins AChE (acetylcholinesterase) and GRIA (Glutamate receptors) have been confirmed to be closely related to chronic CNS disease. It is the target of drugs designed to treat myasthenia gravis, glaucoma, alzheimer's disease, and so forth [70]. GRIA are a heterogeneous family of G-protein-coupled receptors whose function is to modulate brain excitability via presynaptic, postsynaptic and glial mechanisms. Thus, *Rhizoma atractylodis macrocephalae* interacting with these receptors may exert a pharmacological effect on CNS diseases. In addition, KCNA3 (Voltage-gated potassium channel subunit Kv1.3) has been demonstrated that it plays a key role in phenotypes ranging from developmental disorders to adult-onset neurodegeneration [71]. Actually, molecule Isoliquiritigenin (ISL) has been demonstrated actively in the neurodegenerative diseases therapy by binding to KCNA3. ISL protects HT22 hippocampal neuronal cells from glutamate-induced oxidative stress by protect against glutamate-induced neuronal cell death. It is potentially useful in the prevention and treatment of neurodegenerative diseases [72].

In summary, QEH could be a tonic herb to enhance physical strength and endurance, strengthen the immune system, lower blood pressure, promote excretion and enhance normal well-being. Interestingly, QEH cited above have always being prescribed in many frequently used herbal formulaes. For example, Huang Qi Liu Yi Tang (HQLYT, composed of *Radix astragali* and *Radix glycyrrhiz*) is a Chinese medicinal prescription widely used in daily foods to improve health [2]. Clinically, qi-enriching herbs could be adjuvant therapy for chronic phlegmatic disorders including cardiovascular disease, tumors and alzheimer's disease, and so forth.

#### 3.4.2. C-T Network: BTH

In the blood-tonifying conpounds-targets network, there are 44 potential targets which hit by 8 active compounds in all 71 targets related to haematogenous mechanism and detailed information is given in (Table 2).

*Regulation on Hemopoietic Growth Factor. *Hemopoietic growth factors play a key role in hematopoiesis not only by causing differentiation of stem cells toward a particular cell type, but also by inducing the proliferation of cells (i.e., increasing their numbers) and by favoring maturation of the cells (i.e., increasing their function) [79]. Production of these growth factors for clinical use has a significant impact on hematology, oncology and other fields by reducing the morbidity and mortality associated with diseases and treatments. Availability of these growth factors has also expanded the treatment options for many patients, particularly those with malignant neoplasms.

The present work found that 15 targets are connected to induce or regulate hemopoietic growth factor, it has been reported IL-6 (Interleukin-6), TNF (tumor necrosis factor-alpha), SHP-2 (Protein-tyrosine phosphatase 2) and EP4 are responsible for these functions. IL-6 (Interleukin-6) acts synergistically with IL-3 to stimulate increased numbers of granulocyte/macrophage (GM) and multilineage colonies, IL-6 also interacts with all the factors, including M-CSF and GM-CSF, to stimulate an increase in colony size [80]. It has examined TNF (tumor necrosis factor) can promote the growth of erythroid progenitors CFU-E and BFU-E and the hematopoietic cell lines K562, HL60, and HEL cells [81]. In addition, SHP-2 (Protein-tyrosine phosphatase 2) is critical for hematopoietic cell development and function owing to its essential role in growth factor/cytokine signaling. More importantly, germline and somatic mutations in this phosphatase are associated with Noonan syndrome, Leopard syndrome, and childhood hematologic malignancies [82]. And administration of EP4 agonist facilitates the recovery of hematopoietic stem/progenitor cell from 5-fluorouracil (5-FU)-induced myelosuppression, indicating a role for EP4 signaling in this process [83]. Clearly, paeoniflorin which interacts with IL-6 and TNF could promote the proliferation of hematopoietic progenitor cell in bone marrow of radiated mice [4]. Ferulic acid which interacts with SHP-2 and EP4 treatment enhanced hematopoietic progenitor cell activity resulting in accelerated blood cell recovery. Ferulic acid administration can increase the levels of granulocyte-colony stimulating factor (G-CSF) and erythropoietin [84]. These hemopoietic growth factors-related proteins play an important role in promoting hematopoietic progenitor cell proliferation and differentiation into mature blood cells.

*Regulation on Bone Marrow Stromal Cell.* Bone marrow stroma is composed of a variety of different stromal cell providing structural and functional support for hemopoiesis. These matrix components play an active role in control of cell adhesion and migration within the bone marrow, presentation of cytokines in proliferation processes. They act as adhesive substrate for hemopoietic progenitor cells which could help to strengthen the overall binding to the stroma [85].

The present work found that 9 targets are connected to regulate the bone marrow stromal cell, such as ITG (integrin), ITGAV (vitronectin receptor alpha V) and CAMS (cell adhesion molecule). Pre-osteoblasts and their bone marrow stromal cells precursors may use matrix metalloproteinase 2/bone sialoprotein/ITG complexes to disrupt matrix barriers during migration to their final destinations in vivo [86]. Fibronectin binds to the ITGAV specifically with high affinity, and this interaction is biologically relevant in supporting cell adhesion to matrix proteins [87]. CAMS may be responsible for cell adhesion between hematopoietic cells and bone marrow stroma, thus, CAMS is important in maturation or differentiation of normal and neoplastic hematopoietic cell precursors [88]. Thus, we deduce that the functions of hematopoiesis are facilitated by this bone marrow stromal cell regulatory protein.

*Regulate Hematopoietic Stem Cell Apoptosis. *Because most mature blood cells have a very short life span, the importance of haemopoietic stem cells (HSC) in sustaining the life of the mammal is very critical. Self-renewal and apoptosis of HSC represent major factors determine the size of the haemopoietic cell mass. In normal haemopoiesis, these factors are controlled so that steady-state kinetics are preserved. In contrast, in myeloid leukemias, myeloid expansion can be explained by an increase in self-renewal, and reduced apoptosis. Therefore regulating the apoptosis of hemopoietic system could effectively control the process of disease. Clinical studies demonstrated that *Angelica sinensis* extract promotes hematopoiesis and thrombopoiesis in the mouse model. This effect results from the anti-apoptosis activity of *Angelica sinensis *and is likely to involve the PI3K/AKT pathway [89].

Inspecting of the network found that 6 molecules have strong interactions with 14 proteins. Some proteins have been confirmed that they are closely related to hematopoietic stem cell apoptosis. For instance, It has been reported that prostaglandin E2 (PGE2) receptors enhance HSC survival, associated with an increase in survivin mRNA and protein expression and reduction in intracellular active caspase-3 [90]. Bcl-2 family members involved in regulating apoptosis directly affect cell cycle progression. Anti-apoptotic family members, including Bcl-2, can cause exit from, and delay entry into the cell cycle, something that is apparent in hematopoietic stem cell overexpressing Bcl-2. Pro-apoptotic family members, including Bax, have the opposite effect [91]. In addition, Caspases (CASP) are crucial mediators of programmed cell death (apoptosis). Among them, CASP-3 is a frequently activated death protease, catalyzing the specific cleavage of many key cellular proteins [92]. Clearly, catalpol which interacts with Bcl-2 and CASP-3 could significantly inhibit ionizing radiation (IR)-induced human lymphocyte AHH-1 cells apoptosis and increase cells viability in vitro [93]. Moreover, MIF delayed cleavage of the proapoptotic members of the Bcl-2 family Bid and Bax in neutrophils, suggesting that MIF inhibits apoptosis pathways proximal to mitochondria activation [94]. All these are related to proliferation and apoptosis of Hematopoietic cell. Apoptosis of hematopoietic cell is an important component in the development of hemopoietic system, therefore the regulation of these proteins may inhibit the apoptosis of hemopoietic system and further control the process of hematopoiesis.

Hematopoietic abnormality has been observed in patients with aplastic anemia, myeloproliferative diseases, leukemia, myeloma, anaphylactoid purpura, uremia, hemophilia and thrombus. In western medicine, androgen has been widely used as hematopoiesis reconstruction medicine in clinic [95], an increasing number of studies have indicated that androgen may be associated with undesired effects. Usual side effects have been reported mainly in adolescents, young men and women and are consist of flushing of the skin and acne, hirsutism, deepening and hoarseness of the voice, masculinization, amenorrhea and increase in libido [96]. We can disclose the action mechanisms from the network target viewpoint: BTH may regulate hemopoietic growth factor, bone marrow stromal cell and hematopoietic cell apoptosis process to against BDS-related hematopoietic abnormality processes. The present work creates valuable insight on the possible effects and utilization of BTH and its components as a feasible alternative therapeutic agent for patients with hematopoietic disorders or “androgen intolerance”. Interests in TCM from natural sources as a feasible alternative therapeutic agent for the prevention of hematopoietic disorder in patients are growing. For example, Si-Wu-Tang, comprising four medical plants, that is, *Paeonia lactiflora*, *Ligusticum chuanxiong*, *Angelica acutiloba* and *Rehmannia glutinosa* libosch, is one of the most widely used formulae of TCM. It has been used as the hematinic to treat BDS in clinic for hundreds of years in China and Japan (Japanese name, Shimotsuto) [97].

The analysis mentioned above shows that QEH or BTH cannot be separated into independent clusters only according to the target profiles, suggesting that the features of target profiles of the ingredients from different herbs are overlapped. As consistent with the SOM clustering results, QEH have a huge potency to hit massive targets and treat a variety of ailments while targets and curative effect of BTH are relatively single/centralized. Thus, this new subject of TCM system pharmacology is updating the research paradigm from current “one target, one drug” to “network target, multicomponent therapeutics”, which refers to the comprehensive analysis for therapeutic effects of TCM on the basis of the identification of the network target underlying a given disease or TCM Syndrome as well as the target network of a given TCM.

#### 3.4.3. The Relationships between Multicomponents and Synergistic Effects

Synergistic multitarget effects means that natural products affect not only one target, but several targets such as enzymes, substrates, metabolites, receptors, ion channels, transport proteins or DNA/RNA and can cooperate in an agonistic and synergistic way. Synergistic interactions are of vital importance in phytomedicines, to explain rational and efficient therapy form of combinational effects to be greater than the sum of the individual effects. They explain the efficacy of apparently low doses of active constituents in a herbal product [98]. In this regard, QEH and BTH may have the potential of addressing a relationship between multicomponents and drug synergistic effects, thereby increasing the likelihood of conquering complex diseases, such as QEH for cardiovascular diseases. Based on the analysis from the drug-target-disease network of cardiovascular system, the following mechanisms were elucidated:

1. As shown in Figure 3, many potential targets are targeted by more than one candidate compounds. PTGS2, iNOS, MAOB, PTGS1 are examples of highly connected potential targets, whose numbers of candidate compounds are 12, 6, 5 and 5 respectively. The common targets shared by multi-compounds imply that the QEH might exert synergistic therapeutic effects on cardiovascular diseases, which are probably more effective than single compounds.
2. It has been recognized that cardiovascular disease continuum begins with risk factors that initiate the process, leading to tissue damage. Inhibition of an individual target is insufficient to restore the cardiovascular system to the healthy state. In this case, multiple compounds contained in QEH could hit a series of targets, which have been annotated to have significant relationship with the pathological process of cardiovascular disease, and finally exert synergistic therapeutic efficacies. For example, PLA2G4A, MMP9, ALOX15 and TSP1 are closely concerned with atherosclerosis, while PIK3CG, PGF, VCAM1 and ITGA2B are related to myocardial infarction. Proteins ADRB1 and SCN5A may play important roles in arrhythmia. PGF and ADRB1 are concerned with vasoconstriction, the regulation of them may cause hemangiectasis, and then lower blood pressure. Therefore, it can be deduced that the action mechanism of this medical composition is that the QEH systematically controls the cardiovascular disease via potentially synergistic interactions of the active compounds.

## 4. Conclusion 

Traditional Chinese Medicine is a unique (independent) system of theory, diagnosis and treatment tools in terms of composition or from the pharmacodynamics. As we know, different from the western medicine, TCM aims to correct mal-adjustments and restore the self-regulatory ability of the body, and not to antagonize specific pathogenetic targets. TCM does not focus solely on the disease defined by specific pathological changes, but concentrate on the overall functional state of the patient. In TCM theory, disease status is considered as the unbalance of the whole body system, and concoctions of natural products are formulated to regain the balance of the system.

**Figure 5:**
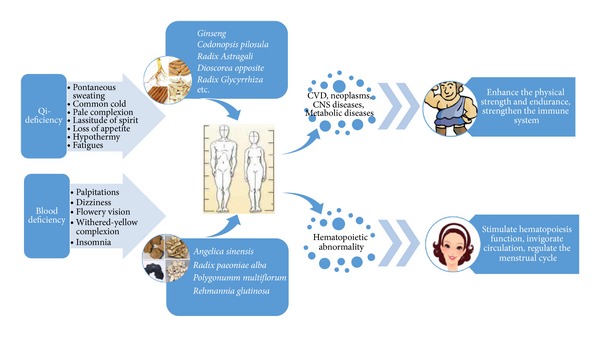
Flowchart shows the process of QEH and BTH application in a simplified sequence. The flowchart suggests QEH enhances physical strength, immune system and normal well-being while BTH stimulates hematopoiesis function in body. As a result, both qi-enriching herbs and blood-tonifying herbs could enhance human normal well-being and increase the overall quality of life.

In this work, we have proposed a new modeling system, combining oral bioavailability screening, pattern recognition, multiple drug targets prediction and validation, network pharmacology to investigate the material basis of representative TCM recipe qi-enriching and blood-tonifying Chinese herbs for the treatment of QDS and BDS. Our research not only makes a better understanding of the mechanisms of blood-tonifying and qi-enriching herbs, but also provides new insights into interpreting the theory of “qi and blood” of TCM. Our main findings are:

1. A model for classification of qi-blood compounds was established, exhibiting proper reliability and high prediction accuracy. By an analysis of those statistically significant descriptors implicated in the model, the interpretation of the models in terms of the chemical features influencing the qi-enriching and blood-tonifying compounds were conducted. In summary, qi-enriching compounds have characteristics of high lipophilicity and C nonsaturated property while blood compounds possess high chemical reactivity.
2. The pharmacodynamic components and the corresponding targets of these qi-enriching and blood-tonifying herbs have been predicted, which provides the desire to seek the mechanisms of action of qi-enriching and blood-tonifying herbs in curing QDS and BDS. Our results demonstrate why the qi-enriching herbs have effectiveness on cardiovascular disease, neoplasms, nervous system diseases, nutritional and metabolic diseases and other diseases while blood-tonifying herbs is works through promoting bone marrow hematopoietic. As a result, both qi-enriching herbs and blood-tonifying herbs could enhance human normal well-being and increase the overall quality of life.
3. Our network-based TCM pharmacology provides a perspective for better understanding of the holistic, complementary and synergic essence of qi-enriching and blood-tonifying herbs which is efficient in treating QDS and BDS at a molecular level. TCM, in essence, is combination therapy by multiple active compounds (Figure 5).

Despite these potentially interesting associations, cautious interpretation is warranted as these findings mainly relied on statistical analysis. Further experimental testing of these hypotheses will be required to support further assessments of potential clinical applications. Hopefully, our systems pharmacology method is extensible to other herbal medicines, which provides a reliable and practical strategy to identify the active herbal ingredients and potential synergistic correlativity, to reveal the mechanisms of botanic drugs, and to facilitate drug discovery as well.

## Supplementary Material

Table S1: This table contains the information of all compounds belong to 8 qi-enriching herbs. It covers qi-enriching TCM name, compounds name in each TCM and their value of Oral Bioavailability and Drug-likeness.
Table S2: This table contains the information of all compounds belong to 4 blood-tonifying herbs. It covers blood-tonifying TCM name, compounds name in each TCM and their value of Oral Bioavailability and Drug-likeness.

## Figures and Tables

**Table 1:** Active compounds of qi-enriching herbs in this work and their targets of different diseases.

Disease types	TCM	Effective molecule	Target	Reference*
Anti-inflammation	*Rhizoma atractylodis macrocephalae *	AtractylenolideI	IL1B, iNOS, PTGS1, PTGS2, SRC	
	*Rhizoma atractylodis macrocephalae *	AtractylenolideIII	iNOS	
	*Radix glycyrrhiza *	Isoliquiritigenin	MIF, PTGS2,	[8]
	*Radix glycyrrhiza *	licochalcone A	iNOS, PTGS1, PTGS2,	
	*Radix glycyrrhiza *	Ononin	ALOX5, CD46, MIF, iNOS, PTGS1, PTGS2, RXRA	
	*Rradix astragali *	Calycosin	ALOX5, MIF, HCK	[6, 7, 42, 73]
	*Rradix astragali *	Isoflavanone	IDO1, PTGS1,	
	*Ginseng *	Ginsenoside-Rb1	IL1B, NFKBIA, PRKCB	[43]
	*Ginseng *	Ginsenoside-Re	iNOS	
	*Ginseng *	Ginsenoside-Rf	IFNG, IL1B, IL4, PTGS2	
	*Ginseng *	Ginsenoside-Rg1	IL2, IL4, PRKCB	
	*Ginseng *	Ginsenoside-Rg3	IFNG, IL1B, IL4, iNOS, PTGS2	
	*Dioscorea opposite *	Batatasin I	HCK	
	*Dioscorea opposite *	Diosgenin-3-O-beta-D-glucopyranoside	PTGS2	
	*Panax quinquefolium *	Pseudostellaria G	GRB2, ITGA4,	

CVD	*Rhizoma atractylodis macrocephalae *	Atractylone	ADRB1, ADRA1A, ADRA1B, SCN5A	
	*Rhizoma atractylodis macrocephalae *	AtractylenolideI	F13A1, ALAD, PGF, VEGFA	
	*Radix glycyrrhiza *	Isoliquiritigenin	SELE^a^, F11R^b^, JAK2^c^, VCAM1, PTGS1,	[53]
	*Radix glycyrrhiza *	licochalcone A	ADRA1B, FOSL2, SCN5A, HDAC5, HDAC9, CHRM1,	
	*Radix glycyrrhiza *	Ononin	KDR, PIK3CG,	
	*Rradix astragali *	Isoflavanone	ALOX15,	
	*Rradix astragali *	Calycosin	PI3K-beta, ABL1, PIK3CG, ALOX15, PIK3CG	
	*Ginseng *	Ginsenoside-Ra1	PTPN2	[52]
	*Ginseng *	Ginsenoside-Rb1	PTPN2, VEGFA, VCAM1	
	*Ginseng *	Ginsenoside-Rb2	PTPN2	
	*Ginseng *	Ginsenoside-Rd	PTPN2	
	*Ginseng *	Ginsenoside-Rg3	MMP9,	
	*Ginseng *	Ginsenoside-Re1	TSP1	
	*Ginseng *	Ginsenoside-Rg1	ACTA2, PTPN2	
	*Dioscorea opposite *	Batatasin I	PI3K-beta, ALOX15	
	*Dioscorea opposite *	Diosgenin-3-O-beta-D-glucopyranoside	HIF1A, VEGFA, PLA2G4A	
	*Panax quinquefolium *	Pseudostellaria G	FNRB, ITGA2B	[74]

Metabolic process	*Rhizoma atractylodis macrocephalae *	Atractylone	PDE3A	
	*Rhizoma atractylodis macrocephalae *	AtractylenolideI	GPT, ADH1C, AR, ASS1, ASPC, GATM, PLA2G2E, IDH1, ME2, PTER, TPI1, PRSS1, PRSS3	
	*Radix glycyrrhiza *	Isoliquiritigenin	PRKACA, PKIA, METAP1, PPAR-gamma	[75]
	*Radix glycyrrhiza *	licochalcone A	CALM, EIF6, GCGR, GSK3B, MAPK14, PPAR-gamma, PRSS1	
	*Radix glycyrrhiza *	Ononin	CALM, PRKACA, PKIA, PDE3A, HSD17B3, GSK3B, GLO1, MCHR1, PPAR-gamma, PRSS1	
	*Rradix astragali *	Calycosin	HSD17B3, GLO1, MCHR1	[76]
	*Ginseng *	Ginsenoside-Rb1	GSK3B	[61]
	*Ginseng *	Ginsenoside-Rg3	PRKAG2, PPAR-gamma	
	*Dioscorea opposite *	Diosgenin-3-O-beta-D-glucopyranoside	HSD17B3, FAS, NR1I2, AKT1, MTOR AKT1, MTOR	[77]
	*Dioscorea opposite *	ergosterol	DHCR7^b^	
	*Panax quinquefolium *	Pseudostellaria G	OPRM1	[78]

Anticancer	*Rhizoma atractylodis macrocephalae *	AtractylenolideI	CTSD, TNF, SRC,	
	*Rhizoma atractylodis macrocephalae *	AtractylenolideIII	TNF	
	*Radix glycyrrhiza *	licochalcone A	CDK4, CCND1^b^, RB1, RELA, CHEK1, STAT3^d^, HDAC9	
	*Radix glycyrrhiza *	Isoliquiritigenin	HDAC1, HDAC2, HDAC3, HDAC5, HDAC9, SLC2A1, FOS^b^	
	*Radix glycyrrhiza *	Ononin	CHEK1, RET	
	*Rradix astragali *	Calycosin	CDK4, TERT, RET, CHEK1	[10]
	*Ginseng *	Ginsenoside-Rf	TNF	[68]
	*Ginseng *	Ginsenoside-Rg1	SMAD2, FN1, TGFB1	
	*Ginseng *	Ginsenoside-Rc	FOS	
	*Ginseng *	Ginsenoside-Re	FOS	
	*Ginseng *	Ginsenoside-Rg3	TNF	
	*Panax quinquefolium *	Pseudostellaria G	CXCR4, HDAC1, HDAC2, HDAC3, HDAC5, HDAC9, ITGA6	
	*Dioscorea opposite *	Batatasin I	PDGFRA	
	*Dioscorea opposite *	Diosgenin-3-O-beta-D-glucopyranoside	CAT, TP53, SHH, RELA	

CNS diseases	*Rhizoma atractylodis macrocephalae *	AtractylenolideI	AChE, BCHE, GABRA1, GLRA1, Nos1, ABAT,	[71]
	*Rhizoma atractylodis macrocephalae *	AtractylenolideII	AChE, GABRA1, GABRA1, GABRA1, GRIA2, CHRNA7	
	*Rhizoma atractylodis macrocephalae *	AtractylenolideIII	AChE, GABRA1, GABRA1, GRIA2, CHRNA7	
	*Rhizoma atractylodis macrocephalae *	Atractylone	DRD1, HTR2A, CHRNA7, SLC6A3, SLC6A2, CHRM1, CHRM2, CHRM3, CHRM4	
	*Radix glycyrrhiza *	Isoliquiritigenin	HSP90AA1, MAOB, KCNA3, APP, MAPK14,	
	*Radix glycyrrhiza *	licochalcone A	HSP90AA1, MAOB, SLC6A3, KCNA3, MPK1, APP,	
	*Radix glycyrrhiza *	Ononin	HSP90AA1, MAOA, MAOB, NQO1, SLC6A2, MAPK14,	
	*Rradix astragali *	Calycosin	MAOA, MAOB, NQO1, CD46	
	*Rradix astragali *	isoflavanone	MAOA, MAOB, NQO1, SIGMAR1, CD46, MT-ND1, MT-ND2, MT-ND3, MT-ND4, MT-ND4L, MT-ND5, MT-ND6	
	*Ginseng *	Ginsenoside-Rb1	PLAT,	
	*Ginseng *	Ginsenoside-Rg3	HTR3A	
	*Panax quinquefolium *	Pseudostellaria G	OPRD1, OPRK1	

*The corresponding efficacy of botanic drug has been supported by the literature.

Superscript with targets: The functional activity of the components with regard to the targets.

^a^Down-regulate gene. ^b^Decrease expression or activity. ^c^Increase expression level or activity. ^d^Directly inhibit.

**Table 2:** Active compounds of blood-tonifying herbs in this work and their targets of different diseases.

Disease	TCM	Molecular	Target	Reference*
Regulate hemopoietic growth factor	*Radix paeoniae alba *	Albiflorin	PKC*α*	
	*Radix paeoniae alba *	Paeonioflorin	IL-6, LBP, PKC*α*, TNF	
	*Angelica sinensis *	Ferulic acid	ADRA2A, PTGER4, SHP-2, F2, TUBB1	
	*Angelica sinensis *	ligustilide	SOD1, TNF^a^	
	*Polygonum multiflorum *	Stilbene glucoside	CALM, F7, F10, F2, NQO2, PTPN1	
	*Rehmannia glutinosa *	Catalpol	NOS2^b^, F2, SOD1	
	*Rehmannia glutinosa *	Verbascoside	PKC*α*, F2	

Regulate bone marrow stromal cell	*Radix paeoniae alba *	Paeonioflorin	CD14	
	*Angelica sinensis *	Ferulic acid	MAOA, MAOB, DPP-IV, ITGB3, ITGB5, MAOB, ITGAV	[15]
	*Angelica sinensis *	ligustilide	ACHE^b^	
	*Polygonum multiflorum *	Stilbene glucoside	DPP-IV	
	*Rehmannia glutinosa *	Catalpol	DPP-IV	
	*Rehmannia glutinosa *	Verbascoside	CAM1	

Regulate hematopoietic stem cell apoptosis	*Angelica sinensis *	Ferulic acid	APP, ADRB2, CDK2, DNA pol beta, MIF, PTGER2, PTGER3, RARG, PIM1, TBXAS1	
	*Angelica sinensis *	ligustilide	BAX^b^, Bcl-2, CASP3^b^	
	*Polygonum multiflorum *	Stilbene glucoside	CDK2, CCNA2, PIM1	
	*Rehmannia glutinosa *	5-Hydroxymethyl-furoic acid	CDK2	[17]
	*Rehmannia glutinosa *	Catalpol	Bcl-2^c^, CASP3^b^	
	*Rehmannia glutinosa *	Verbascoside	APP	

*The corresponding efficacy of botanic drug has been supported by the literature.

Superscript with targets: The functional activity of the components with regard to the targets.

^a^Down-regulate gene. ^b^Decrease expression or activity. ^c^Increase expression level or activity. ^d^Directly inhibit.
